# Dementia in elderly patients undergoing early cholecystectomy for acute cholecystitis: a retrospective observational study

**DOI:** 10.1186/s12893-019-0548-y

**Published:** 2019-07-01

**Authors:** Yasunori Nishida, Noriaki Otagiri, Seijiro Yoshifuku, Kenji Misawa, Kenju Ko, Kotaro Sasahara, Hirofumi Kishimoto, Osamu Mishima, Katsunori Tauchi

**Affiliations:** Department of Surgery, Aizawa Hospital, 2-5-1 Honjou Matsumoto, Nagano, 390-8510 Japan

**Keywords:** Acute cholecystitis, Cholecystectomy, Dementia, Aged, 80 and over, Emergency medicine

## Abstract

**Background:**

Dementia often adversely affects postoperative outcomes in surgical patients. This study evaluated postoperative outcomes among elderly patients with and without dementia undergoing early cholecystectomy for acute cholecystitis (AC).

**Methods:**

A total of 182 patients over 85 years of age who were diagnosed with AC and treated from January 2005 to March 2018 were reviewed retrospectively; 59 patients who underwent early cholecystectomy were enrolled. The complication rates, length of postoperative hospital stay, and rates of routine discharge (i.e., returning to their preoperative living location) were compared between two groups of patients with and without dementia.

**Results:**

The overall complication rate after early cholecystectomy for AC in 59 patients was 11.9%, and there was no mortality in this series. The median postoperative hospital stay was 9.0 days, and the routine discharge rate was 89.8%. Of the 59 patients, 22 patients (37.3%) had a history of dementia. Complication rates were comparable between the groups, despite the rate of delirium development being significantly higher in the dementia group. The median length of postoperative hospital stay and routine discharge rates did not significantly differ between groups.

**Conclusions:**

Early cholecystectomy for patients with AC over 85 years of age was performed safely, and elderly patients with dementia had similar postoperative outcomes as compared with patients without dementia.

## Background

Acute cholecystitis (AC) is a disease that usually requires emergency intervention, because it can lead to a number of serious and potentially fatal complications without treatment. To treat AC, the Tokyo Guidelines recommend early cholecystectomy soon after onset [1, 2]. In young and otherwise healthy patients, early cholecystectomy is accepted as the gold standard of treatment for AC. However, in elderly patients (i.e., over 65 years of age), whether early cholecystectomy should be conducted as the first-line treatment for AC is still debated, given their various comorbidities, decreased systemic condition, and/or cognitive impairment.

Dementia is a progressive and life-limiting condition characterized by decreased cognitive function that interferes with daily life. Advanced age is a significant risk factor for the development of dementia [3], and the global population of patients with dementia is estimated to double every 20 years, totaling 81.1 million people by 2040 [4]. When undergoing general anesthesia and surgical interventions, patients with dementia are at a high risk of developing postoperative complications that can lead to a prolonged hospital stay [5, 6]. Moreover, a history of dementia also affects the decision for surgery for patients, their families, and surgeons [7].

The rate of AC in elderly people is expected to increase exponentially. However, no studies have evaluated the association between dementia and outcomes after early cholecystectomy for AC in elderly patients. Therefore, this study aimed to evaluate postoperative outcomes among elderly patients with and without dementia undergoing early cholecystectomy for AC.

## Methods

A retrospective review was performed among patients over 85 years of age who were diagnosed with AC and treated at Aizawa Hospital from January 2005 to March 2018. The data of the patients who underwent early cholecystectomy were evaluated. The study approval and a waiver of informed consent were obtained from the Institutional Review Board of Aizawa Hospital (#2018–037).

Regardless of older age and impaired cognitive status, AC is considered to be an indication for emergency surgery in our department, if patients are recommended for early cholecystectomy as the first-line treatment of AC by the emergency room assessment based on the Tokyo Guidelines [1, 2]. Conservative treatment and biliary drainage are conducted if patients are unable to undergo anesthesia or surgery because of low performance status and/or serious comorbidities. After agreement for the indicated treatment by patients and/or their family, cholecystectomy is conducted at the earliest possible opportunity. In this study, early cholecystectomy was defined as upfront cholecystectomy within 72 h after symptom onset. Generally, laparoscopic cholecystectomy represents the standard of care for AC in our department. Open cholecystectomy is performed upon the surgeon’s choice if there is severe inflammation and/or the patient is in poor general condition, making laparoscopic surgery difficult. Antibiotics with second-generation cephalosporin are administered intravenously before surgery and every 12 h after surgery for 48 h.

Data collected from the patients included patient demographics, comorbidities, the American Society of Anesthesiologists (ASA) classification score [8] recorded by preoperative anesthesia evaluation, laboratory data, severity grading of AC, surgical characteristics, postoperative complications, delirium, and postoperative hospital stay. Comorbid conditions were scored using the Charlson comorbidity index [9]. Severity grading of AC conformed to the Tokyo Guidelines [10]. The postoperative complications were scored using the Clavien-Dindo classification [11]. In addition, the patient’s living location before hospitalization and discharge destination were evaluated as a treatment outcome. Routine discharge was defined as a condition wherein the postoperative living location of the patient was the same as their preoperative living location, and otherwise it was defined as non-routine (e.g. transferred to a short-term hospital or a facility). Regarding the diagnosis of dementia, because cognitive assessment using a systematic approach was not performed in emergency settings, only those patients with a documented history of dementia (Alzheimer’s disease, vascular dementia, or dementia with Lewy bodies) were diagnosed as having dementia.

The data generated were analyzed using the IBM SPSS Statistics 21 software package (SPSS Inc., Tokyo, Japan). The clinical characteristics and perioperative outcomes were compared between the two groups with and without dementia using a chi-square analysis for non-continuous variables and the t-test or the Mann-Whitney U test for continuous variables. All the reported *P* values are two-sided, and *P* < 0.05 denoted statistical significance.

The primary goal was to evaluate postoperative outcomes (complication rates, the length of postoperative hospital stay, and routine discharge rates) among elderly patients with and without dementia undergoing early cholecystectomy for AC. The secondary goal was to evaluate the safety of this treatment for elderly patients.

## Results

During the study period, a total of 182 patients over 85 years of age were diagnosed with AC and treated. Of these patients, 59 patients (32.4%) underwent early cholecystectomy; the other 123 patients were treated with non-surgical methods. Of the 59 patients, 22 patients (37.3%) had a preoperative history of dementia.

Patient characteristics are shown in Table 1. The median patient age was 87 years, and 26 patients (44.1%) were male. Dementia was more common in older female patients. Patients in the dementia group had a lower body mass index (20.4 kg/m^2^ vs. 23.2 kg/m^2^; *p* = 0.011) and higher mean Charlson comorbidity index score than those without dementia (1.91 vs. 1.03; *p* = 0.016). No differences in the ASA scores and presence or absence of anticoagulant therapy were seen between the two groups.

**Table 1:** Comparison of preoperative characteristics between dementia and non-dementia group in patients over 85 years of age with acute cholecystitis

	Total (*n* = 59)	Patients with dementia (*n* = 22)	Patients without dementia (*n* = 37)	*p* value
Age, years*	87 (85–100)	89 (85–100)	86 (85–96)	< 0.001
Sex (Male), n (%)	26 (44.1)	6 (27.3)	20 (54.1)	0.045
BMI, kg/m^2^	22.1 (3.8)	20.4 (2.8)	23.2 (4.0)	0.011
Comorbidity, n (%)				
Congestive heart failure	5 (8.5)	3 (13.6)	2 (5.4)	0.264
Cerebrovascular disease	10 (16.9)	6 (27.3)	4 (10.8)	0.103
Chronic pulmonary disease	4 (6.8)	1 (4.5)	3 (8.1)	0.521
Liver disease	2 (3.4)	1 (4.5)	1 (2.7)	0.611
Diabetes	8 (13.6)	1 (4.5)	7 (18.9)	0.120
Chronic kidney disease	3 (5.1)	1 (4.5)	2 (5.4)	0.690
Malignant tumor	2 (3.1)	1 (4.5)	1 (2.7)	0.611
Charlson comorbidity index score	1.36 (1.37)	1.91 (1.23)	1.03 (1.36)	0.016
ASA score, n (%)				0.384
2E	44 (74.6)	15 (68.2)	29 (78.4)	
3E	15 (25.4)	7 (31.8)	8 (21.6)	
Anticoagulant therapy, n (%)	16 (27.1)	7 (31.8)	9 (24.3)	0.531

*BMI* Body mass index, *ASA* American Society of Anesthesiologists

Continuous parameters are presented as the mean (standard deviation) or median (range)*, and categorical parameters are presented as n (%)

Laboratory data and severity grading of AC are shown in Table 2. The severity of AC was graded as Grade I (mild AC) in 17 patients (28.8%), Grade II (moderate AC) in 38 patients (64.4%), and Grade III (severe AC) in 4 patients (6.8%). The rate of Grade II AC in the patient group with dementia was significantly higher than that in the group of patients without dementia (86.4% vs. 51.4%; *p* = 0.007).

**Table 2:** Comparison of laboratory data and severity grading of acute cholecystitis between dementia and non-dementia group in patients over 85 years of age

	Total (*n* = 59)	Patients with dementia (*n* = 22)	Patients without dementia (*n* = 37)	*p* value
Laboratory value				
Serum albumin, g/L	3.48 (0.53)	3.29 (0.55)	3.59 (0.50)	0.041
Total bilirubin, mg/dL*	1.4 (0.3–9.6)	1.6 (0.3–4.5)	1.2 (0.4–9.6)	0.140
Serum creatinine, mg/dL*	0.80 (0.4–6.2)	0.87 (0.5–6.2)	0.79 (0.4–4.6)	0.621
White blood cell, /mm^3^	12,622.3 (5617.3)	14,251.5 (6728.5)	11,634.9 (4661.6)	0.151
Platelets, 10^4^/mm^3^	17.7 (4.98)	16.3 (4.50)	18.5 (5.15)	0.206
C-reactive protein, mg/dL*	9.8 (0.0–39.1)	14.0 (0.0–39.1)	8.6 (0.2–29.1)	0.397
PT-INR*	1.15 (0.92–1.96)	1.21 (0.92–1.96)	1.11 (0.93–1.71)	0.059
TG severity grading, n (%)				0.007^a^
Grade I	17 (28.8)	2 (9.1)	15 (40.5)	
Grade II	38 (64.4)	19 (86.4)	19 (51.4)	
Grade III	4 (6.8)	1 (4.5)	3 (8.1)	

*PT-INR* International normalized ratio of prothrombin time, *TG* Tokyo Guideline

Continuous parameters are presented as the mean (standard deviation) or median (range)*, and categorical parameters are presented as n (%)

^a^ TG severity grading Grade I vs Grade II

The median time from arrival at the hospital to surgery was 6.0 h. Only 5 patients took over 24 h to arrive at surgery, and others mostly arrived within 12 h. Operative characteristics are shown in Table 3. Laparoscopic cholecystectomy was conducted on 38 patients (64.4%), of whom 5 patients were converted to open surgery intraoperatively. No significant differences in the type of operation, operation time, estimated blood loss, or need for transfusion were seen between the two groups.

**Table 3:** Comparison of operation characteristics between dementia and non-dementia group in patients over 85 years of age with acute cholecystitis

	Total (*n* = 59)	Patients with dementia (*n* = 22)	Patients without dementia (*n* = 37)	*p* value
Time to surgery from hospital arrival, hours*	6.0 (3–70)	5.5 (3–70)	7.0 (4–51)	0.089
Operative procedure, n (%)				
Laparoscopic surgery (initially)	38 (64.4)	12 (54.5)	26 (70.3)	0.223
Laparoscopic-open conversion	5	2	3	–
Laparoscopic surgery	33 (55.9)	10 (45.5)	23 (62.2)	0.211
Operation time, min	141.0 (45.5)	128.2 (34.9)	148.7 (49.8)	0.066
Estimated blood loss, mL*	100.0 (3.0–910.0)	100.0 (3.0–910.0)	80.0 (3.0–760.0)	0.349
Transfusion, n (%)	4 (6.8)	2 (9.1)	2 (5.4)	0.479

Continuous parameters are presented as the mean (standard deviation) or median (range)*, and categorical parameters are presented as n (%)

The postoperative outcomes and their relationships with the dementia group and the non-dementia group are shown in Table 4. Postoperative complications (Clavien-Dindo classification grade > 1) occurred in 7 patients (11.9%), and there was no mortality in this study series. The postoperative complications included cholangitis (*n* = 1), surgical site infection *(n* = 4), intra-abdominal abscess formation (*n* = 1), and deep vein thrombosis (*n* = 1). The median postoperative hospital stay was 9.0 days. No differences in postoperative complications and hospital stay were seen between the two groups; however, postoperative delirium occurred more frequently in the dementia group (63.6% vs. 18.9%; *p* = 0.001). Regarding living location before and after hospitalization, 7 patients (11.9%) were discharged non-routinely, whereas 53 patients (89.8%) were discharge to the same preoperative location. Thus, routine discharge rates were not significantly different between patients with and without dementia.

**Table 4:** Comparison of postoperative outcomes between dementia and non-dementia group in patients over 85 years of age with acute cholecystitis

	Total (*n* = 59)	Patients with dementia (*n* = 22)	Patients without dementia (*n* = 37)	*p* value
Postoperative complications (Clavien grade > 1), n (%)	7 (11.9)	3 (13.6)	4 (10.8)	0.525
Delirium, n (%)	21 (35.6)	14 (63.6)	7 (18.9)	0.001
Postoperative hospital stay, days*	9.0 (4–57)	12.0 (4–37)	7.0 (4–57)	0.079
Routine discharge, n (%)	53 (89.8)	18 (81.8)	35 (94.6)	0.131

Continuous parameters are presented as median (range)*, and categorical parameters are presented as n (%)

Routine discharge was defined as a condition wherein discharge disposition was the same as the preoperative destination

Among 123 patients over 85 years of age with non-surgical treatment, dementia was identified in 51.8%, and no difference in the length of hospital stay was seen between two groups of patients with and without dementia (16.0 vs. 17.0 days, median; *p* = 0.489).

## Discussion

This study demonstrated that an early cholecystectomy for patients with AC over 85 years of age could be performed safely, and elderly patients with dementia had similar postoperative outcomes concerning complication rates, postoperative hospital stay, and routine discharge rates as compared with patients without dementia.

Generally, dementia often adversely affects postoperative outcomes in surgical patients. [5, 6]. Patients with dementia have a higher number of comorbidities than those without dementia, associated with a higher incidence of postoperative adverse events. Hu and colleagues performed a large-scale cohort study on digestive surgery that revealed that the incidence of postoperative complications and mortality rates were higher, and the postoperative length of stay was longer in patients with dementia [6]. However, they included every type of surgery in their study, and detailed analysis focusing on specific procedures was not performed. The present study focused only on early cholecystectomy for elderly patients with AC, and no difference was observed in the incidence of postoperative complications, postoperative length of stay, and rate of routine discharge between elderly patients with and without preoperative dementia, despite higher Charlson comorbidity index scores in patients with dementia. These results suggest that the beneficial effects for patients undergoing early cholecystectomy for AC are significant, and patients have a rapid recovery from the morbid condition due to AC. The impact of dementia on postoperative outcomes might differ according to the severity of the disease and the details of the surgical procedures.

In this study, postoperative delirium occurred in 63.6% of dementia patients, and this rate was higher than that seen in those without dementia. Some authors previously suggested that about 10–50% of elderly patients undergoing surgical treatment might develop delirium postoperatively, which was associated with adverse events and detrimental outcome [12, 13]. Elderly patients need a different level of perioperative care than younger patients. The perioperative care of elderly patients should involve a multidisciplinary team of health professionals, including the surgeon or geriatrician, physiotherapist, and a social worker.

As described in the Tokyo Guidelines [1, 2], early cholecystectomy is the gold-standard treatment for AC, if patients meet the criteria of the preoperative assessment. However, the optimal treatment strategy for elderly patients with AC has not been determined. Loozen and colleagues demonstrated in their systematic review that early cholecystectomy for AC in patients over 70 years of age was associated with a perioperative mortality of 3.5% and morbidity of 24%, and concluded that early cholecystectomy seemed to be a feasible treatment for AC in elderly patients [14]. In the present study, patients over 85 years of age were examined, and the median postoperative hospital stay was 9.0 days with no mortality and low morbidity (11.9%), which we considered nearly acceptable. However, to assess the advantages of upfront surgery for elderly patients with AC, activities of daily life and quality of life are especially important items with regard to the outcomes of surgical treatment [15]. In this study, discharge disposition was assessed for the evaluation of patients’ activities, and the routine discharge rate was 88.1%.

This study was limited by several factors. First, the definition and severity level of cognitive insufficiency were not determined accurately because of the retrospective nature of this study. Therefore, we used only the documented diagnosis of dementia to include demented patients carefully, whereas patients with mild dementia were potentially included in the non-dementia group. Second, in our department, early cholecystectomy is usually performed for almost all patients with AC. The management of conservative treatment and biliary drainage for patients without receiving surgery was not standardized. Therefore, whether early cholecystectomy is superior to non-surgical treatment for elderly patients with AC was not determined in this study. However, the mortality and morbidity rate after early cholecystectomy were 0.0 and 11.9%, respectively, which were acceptable considering previous studies on other treatment strategies for AC in elderly patients [16, 17]. Further prospective studies are needed to reveal the preoperative cognitive status and the efficacy of the surgical treatment for elderly patients with AC. Despite these limitations, our findings should be valuable to surgeons in deciding the surgical indication for AC in elderly people in the emergency room.

## Conclusions

Early cholecystectomy for patients with AC over 85 years of age was performed safely, and elderly patients with dementia had similar postoperative outcomes concerning complication rates, postoperative hospital stay, and routine discharge rates as compared with patients without dementia.

## Data Availability

Please contact the author for data requests.

## Abbreviations

AC: Acute cholecystitis
ASA: American Society of Anesthesiologists
BMI: Body mass index
PT-INR: International normalized ratio of prothrombin time
TG: Tokyo Guideline
